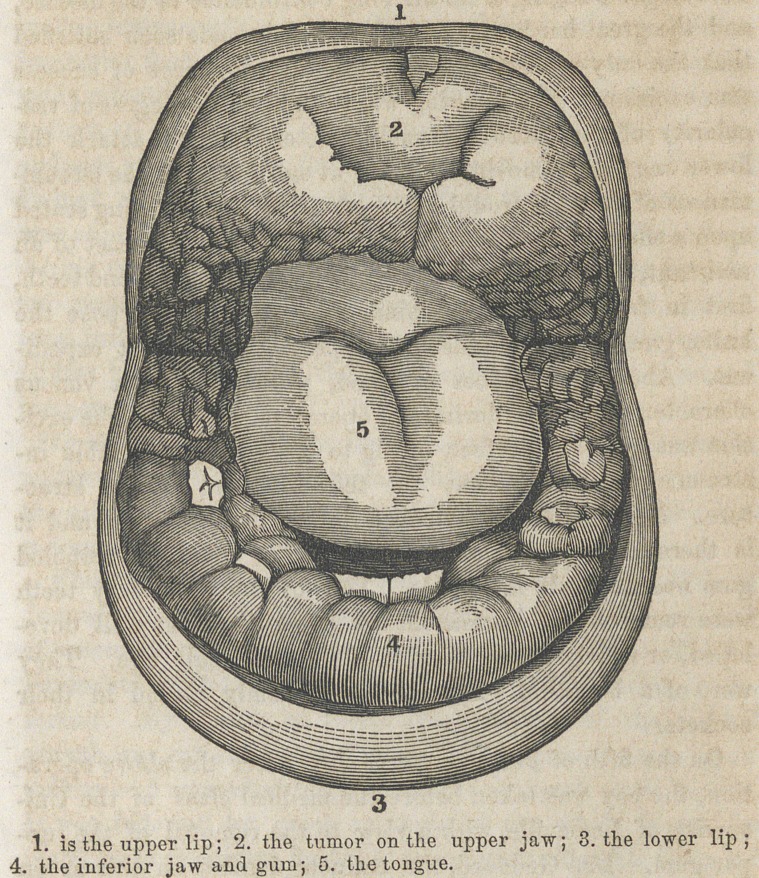# Case of Hypertrophy of the Gums, in the Practice of Professor Gross

**Published:** 1856-04

**Authors:** William H. Goddard

**Affiliations:** Louisville, Ky.


### CASE OF HYPERTROPHY OF THE GUMS,

In the practice of Professor GROSS. Reported by WILLIAM H. GODDARD, D. D. S., Louisville, Ky.

Sometime in December, 1855, I was requested by Dr. Gross, Professor of Surgery in the University of Louisville, to call at St. Joseph's Infirmary in this city, to see a lad who was afflicted with a remarkable disease of the gums, in order to take an impression of his mouth.

On looking at him he presented the appearance as if his tongue were protruding beyond the jaws; but which, upon more careful inspection, was found to proceed from the affected gums, which, projecting upwards and outwards, forced the upper lip against the nostrils, and greatly impeded respiration. Upon examining the mouth, I soon discovered that I should have great difficulty in distending it sufficiently to get in my wax, but after several attempts I succeeded in obtaining a fair impression of the posterior part of the upper jaw and roof of the mouth. Trimming the wax in front and on the top of the gum, I replaced it in the mouth, and took the front impression of the same jaw, making thus two separate pieces. The lower jaw gave me no trouble.

I send herewith the impressions taken before and after the operation, and will thank you to place them in the cabinet of the Ohio Dental College.

I am indebted to my friend, Professor Gross, for the subjoined sketch of this case, copied from his manuscript. He will himself publish a more elaborate history of it in the July number of the Louisville Medical and Surgical Review, a new journal which is about to be issued in this city, and of which he will be one of the editors.

"The subject of this remarkable case was James W. Outland, aged ten years, a native and resident of Calloway county, Kentucky. The enlargment of the gums was first perceived when he was nine months old, though, from the history of the case, there is great probability that it had existed from his birth; for it had already, when the attention of the mother was first attracted to it, acquired some bulk. About this time an abundant flow of saliva commenced, so irritating as to erode the chin and other parts with which it came in contact. This continued, without any material interruption, for several years, when, by degrees, it ceased, the secretion diminishing sensibly in quantity, as well as losing its acrid qualities. Almost simultaneously with the enlargment of the gums was observed an enlargment of the tonsils, leading to embarrasment of breathing, which has remained up to the present time. Both affections gradually but slowly increased, the general health, meanwhile, being good. About the fifteenth month the two middle incisors of the lower jaw appeared, and after sometime the corresponding teeth above. The gum, however, was already so large as to conceal nearly the whole of these organs. By and by the inferior deciduous cuspid and first molar of the right side issued, and these were the only teeth that were ever visible, except those represented in the cut, prior to the operation performed for the boy's relief.

The condition of the boy when he was brought to me on the 25th of December, 1855, by my friend Dr. Lynch of New Concord, Kentucky, was as follows:

His body was short but thick set, his eyes black, the pupils dilated, and the lids fringed with long lashes, the hair dark, the face pale and pasty, the head rather ill-shaped, and the abdomen preternaturally prominent. The extremities were cold, the bowels regular, and the appetite good. The respiration was embarrassed, and performed with a kind of croaking sound. At night he snored very loud, and often waked up struggling for breath, the voice being muffled, and the articulation indistinct. His pulse was natural, his strength was rather impaired, and his muscles were thin and flabby.

Projecting from the anterior part of the upper jaw was a tumor, of a pale color, inelastic, perfectly insensible, and of firm consistence, presenting very much the appearance of the snout of a hog, as represented in the annexed sketch. It stood off very obliquely, and received but a very partial covering from the corresponding lip. It was rough on the surface, and was about an inch and a quarter in its antero-posterior diameter, its length having been about one inch and a half. At its free margin, which was quite irregular, was seen the tip of the left central incisor. Extending back from this tumor, on each side, the whole length of the jaw, was the enlarged gum, forming a thick, broad ridge, completely embedding the teeth. At several points, particularly behind, the morbid growth was more than nine lines in width; in front and at the middle it was less. It was of a more florid color than the main tumor, but of about the same degree of consistence. Opposite the site of the bicuspid teeth, on each side, it exhibited a remarkably granulated appearance, the excresences having a pedunculated form, and being folded upon each other. Projecting toward the roof of the mouth, it greatly encroached upon this cavity, lessening its capacity, and thus interfering with its functions, as well as with speech and respiration.

The lower gum was in the same condition as the upper, equally hard and insensible, but less developed. It was of a bluish florid complexion, and larger in front and behind than at the intermediate points; its free surface was uneven and so prominent as to hide all the teeth, except the central incisors, the point of the right cuspid, and the cusps of each deciduous and first permanant molars. The appearances here described are admirably represented in the accompaning cut.

1. is the upper lip; 2. the tumor on the upper jaw; 3. the lower lip; 4. the inferior jaw and gum; 5. the tongue.

On looking into the fauces both tonsils were found to be much enlarged, the left, however, more than the right, and the arches of the palate were in a state of chronic inflammation. The uvula was also somewhat hypertrophied. As the respiration was much embarrassed by the state of the tonsils. I excised the redundant portion of the left of these organs as a prelimiary step to the treatment of the other and more important affection.

Various plans of treatment had been employed for the relief of the boy before he came under my charge, but all without advantage. Judging from the long continuance of the disease, and the great hardness of the parts, I became soon satisfied that the only remedy that held out any prospect of success was excision. Not knowing what might be the degree of vascularity of the morbid growth, I determined to attack the lower one first, removing as much at one sitting as the circumstances of the case would admit of. The patient being seated upon a chair, with the head supported against the breast of an assistant, I pared away the gum closely from the jaw and teeth, first in front and then behind, using for this purpose the knife, gum-lancet, or chisel, as appeared to be most expedient. About five ounces of blood, principally of a venous character, were lost during the operation. Behind, the excision was not very perfect, owing to the want of suitable instruments, to scrape away the thickened and callous structure. No pain was experienced during the operation, and it is therefore reasonable to suppose that the hypertrophied gum was perfectly insensible. Two of the temporary teeth were removed, the permanent set being found as well developed, or nearly so, as usual in children at this age. They were of a beautiful white color, and firmly rooted in their sockets.

On the fifth of January, three days after the above operation, the boy was taken before the medical class of the University of Louisville, with a view to the removal of the upper gum. Dr. Goddard, the eminent dentist, on this as on the former occasion gave me his valuable assistance. By means of a chisel, slightly curved, and not very sharp, the greater portion of the morbid mass was easily pared off in front, exposing the permanent incisors, the two central of which were found to be fully developed, while the lateral incisors were only about one third grown. In excising the gum from the posterior surface of the jaw bone in front the anterior palatine artery of the right side was unavoidably laid open, and bled considerably before the flow could be arrested. The larger pieces of the gum being thus removed, Dr. Goddard cut and scraped off the remainder by means of scaling instruments, such as those used by dentists in removing tartar from the teeth, and which were found to be admirably adapted to the object. The proceeding was necessarily tedious on acccount of the frequent spitting of the patient, and was attended with the loss of at least six ounces of blood. Indeed, the lad became quite pale from this cause. No pain was experienced. One of the diciduous teeth was forced out during the operation. The lad now has in his upper jaw, the two central incisors and first molars fully developed, the lateral incisors about one third grown, the cusps of the second molars just visible, and the temporary cuspids and second temporary molars.

In the lower jaw are the four incisors and first permanent molars. The left second molar about one-fourth grown, and the other molar just emerging, with the right temporary cuspid and left temporary molar.

Finally, a third operation was performed on the 17th of January, mainly by Dr. Goddard, consisting in the removal of remnants of gum between and around the teeth. The instruments employed on the occasion were the same as on the former. By means of these every vestige of the morbid growth was cut and scraped away. The operation was again tedious and somewhat bloody, but not as much so as before. It was followed by great soreness of the whole mouth, by inability to masticate food, and even by considerable difficulty of deglutition, continuing for nearly a whole week. In consequence of these occurrences free use was obliged to be made of anodynes, especially at night. Since the last two operations the boy has been taking sulphate of iron and quinine, to improve his general health and the condition of the blood, which, as evidenced by the pallor of his countenance, has been much impoverished by the repeated losses sustained in the excision of the gums. He left Louisville on the 4th of March, still rather pale and feeble, but in fine spirits and with a good appetite. A few days before his departure I excised a portion of the right tonsil, the left, as already stated, having been removed a few days after his arrival in town. His breathing is still too loud, though he no longer snores and perspires during sleep. Orders were given to keep up the steady use of his tonics, to make him exercise freely in the open air, and to put him upon a plain but nutritious diet. The tonsils and arches of the palate are to be mopped every fourth day with a pretty strong solution of nitrate of silver. The teeth are covered up to their necks with healthy gum; and, thus far, there is not the slightest appearance of a return of the morbid growth.

On closing the jaws so as to bring the grinders in contact, a space is found to exist between the front teeth, three quarters of an inch in extent. The upper lip is gradually regaining its proper position, and the contour of the face is greatly improved."

## Figures and Tables

**Figure f1:**
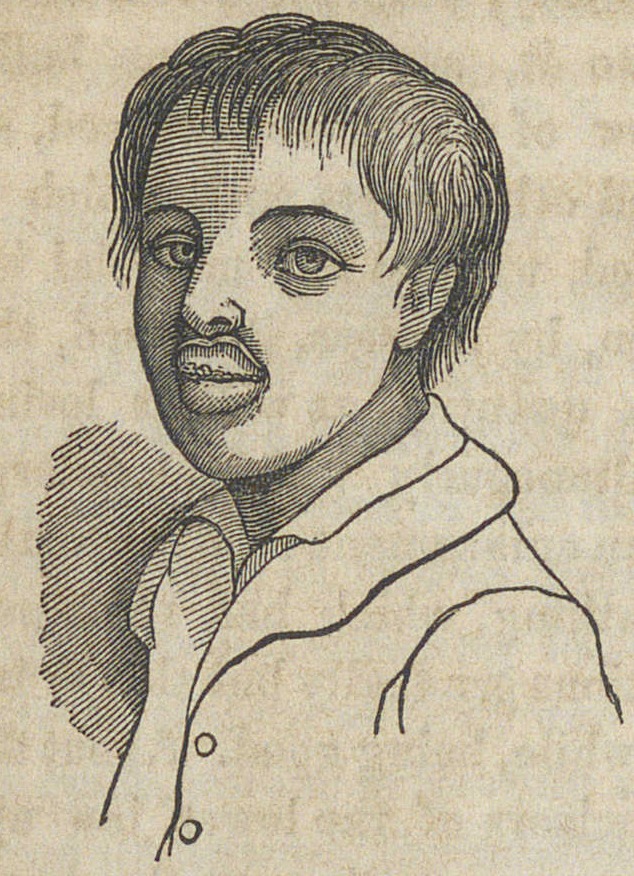


**Figure f2:**